# Biologically Active TNIIIA2 Region in Tenascin-C Molecule: A Major Contributor to Elicit Aggressive Malignant Phenotypes From Tumors/Tumor Stroma

**DOI:** 10.3389/fimmu.2020.610096

**Published:** 2020-12-09

**Authors:** Takuya Iyoda, Motomichi Fujita, Fumio Fukai

**Affiliations:** ^1^ Department of Pharmacy, Faculty of Pharmaceutical Sciences, Sanyo-Onoda City University, Sanyo-Onoda, Japan; ^2^ Department of Molecular Patho-Physiology, Faculty of Pharmaceutical Sciences, Tokyo University of Science, Noda, Japan

**Keywords:** tenascin-C, TNIIIA2, β1-integrin, macrophage, solid tumor, glioblastoma, PDGF

## Abstract

Tenascin (TN)-C is highly expressed specifically in the lesions of inflammation-related diseases, including tumors. The expression level of TN-C in tumors and the tumor stroma is positively correlated with poor prognosis. However, no drugs targeting TN-C are currently clinically available, partly because the role of TN-C in tumor progression remains controversial. TN-C harbors an alternative splicing site in its fibronectin type III repeat domain, and its splicing variants including the type III-A2 domain are frequently detected in malignant tumors. We previously identified a biologically active region termed TNIIIA2 in the fibronectin type III-A2 domain of TN-C molecule and showed that this region is involved in promoting firm and persistent cell adhesion to fibronectin. In the past decade, through the exposure of various cell lines to peptides containing the TNIIIA2 region, we have published reports demonstrating the ability of the TNIIIA2 region to modulate distinct cellular activities, including survival/growth, migration, and invasion. Recently, we reported that the signals derived from TNIIIA2-mediated β1 integrin activation might play a crucial role for inducing malignant behavior of glioblastoma (GBM). GBM cells exposed to the TNIIIA2 region showed not only exacerbation of PDGF-dependent proliferation, but also acceleration of disseminative migration. On the other hand, we also found that the pro-inflammatory phenotypic changes were promoted when macrophages are stimulated with TNIIIA2 region in relatively low concentration and resulting MMP-9 upregulation is needed to release of the TNIIIA2 region from TN-C molecule. With the contribution of TNIIIA2-stimulated macrophages, the positive feedback spiral loop, which consists of the expression of TN-C, PDGF, and β1 integrin, and TNIIIA2 release, seemed to be activated in GBM with aggressive malignancy. Actually, the growth of transplanted GBM grafts in mice was significantly suppressed *via* the attenuation of β1 integrin activation. In this review, we thus introduce that the TNIIIA2 region has a significant impact on malignant progression of tumors by regulating cell adhesion. Importantly, it has been demonstrated that the TNIIIA2 region exerts unique biological functions through the extremely strong activation of β1-integrins and their long-lasting duration. These findings prompt us to develop new therapeutic agents targeting the TNIIIA2 region.

## Introduction

Tenascin (TN)-C, a cell adhesion-modulatory matricellular protein, is characterized by its unique expression pattern. TN-C is transiently highly expressed during embryogenesis, wound healing, and tumorigenesis, whereas its expression in normal tissue is relatively low (1–5). Notably, TN-C is frequently found in most malignant tumors at high levels, with its expression showing a positive correlation with poor disease-free survival in patients with different cancers, such as lung and breast carcinomas and glioma (4, 5). Nonetheless, the role of TN-C in malignant tumor progression remains largely unclear.

As criteria for evaluating tumor cell malignancy, several cellular activities are frequently examined such as proliferation, survival, migration, and differentiation. Any of these cellular activities are strongly linked to cell adhesion. However, the effect of TN-C on cell adhesion control is particularly complex. This is because the TN-C substrate supports the attachment of some cell types but is non-adhesive for others (6–8). Therefore, TN-C is classified as an “adhesion modulatory” ECM protein and is called a “matricellular” protein (9, 10). Because of this antithetical property in cell adhesion control, understanding the role played by TN-C in pathological events is complicated and difficult. 　

**Figure 1:**
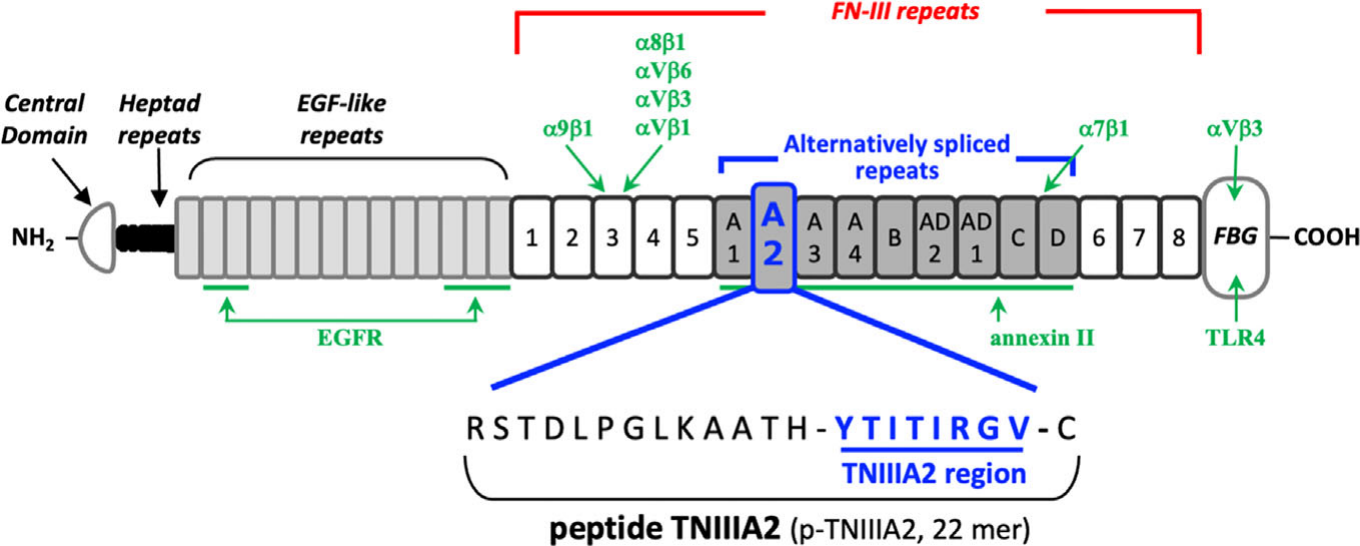
Schematic illustration of the domain structure of tenascin-C and its intramolecular bioactive region TNIIIA2. The sites responsible for receptor binding or ligand binding are shown in green arrow, with the name of corresponding interaction molecules in green text.

TN-C has various isoforms, which are generated by alternative mRNA splicing within its fibronectin type III-like repeat (FNIII) domain A1 to D. The bioactivities of each TN-C isoform are thought to depend on the domains included. Because frequent detection of TN-C containing the FNIII-A2 domain has been reported within tumor-associated pathological lesions (11, 12), it has been presumed that the FNIII-A2 domain may play a role in tumor formation and progression. About 15 years ago, we found a biologically active region in the FNIII-A2 domain of TN-C (**Figure 1**) and gave the responsible 8-amino acid sequence YTITIRGV the name TNIIIA2 (13). This region appeared to be cryptic but was exposed by MMP-2/-9–mediated processing of TN-C (13–15). It has been shown that the released TNIIIA2 region is capable of enhancing cell adhesion to fibronectin (FN) through induction of β1 integrin activation.

Integrin superfamily members act as adhesive transmembrane receptors mediating the cell-to-ECM interaction. Integrins can alter ligand binding and signal transduction activities. Given that integrin-mediated cell-to-ECM interactions play key roles in the induction of appropriate cellular functions in normal cells, including macrophages, changes in the affinity or specificity of the interaction between integrins on the cell surface and the surrounding ECM might be critical for anchorage-dependent cellular processes. Because tumor malignancy is characterized by abnormalities in cell survival, growth, and migration, all of which involve anchorage-dependent cellular processes, there is a possibility that aberrant β1 integrin activation induced by the TNIIIA2 region might be a trigger for eliciting malignancy from tumors and/or the tumor stroma.

In this review, we introduce our recent observations concerning the effect of the TNIIIA2 region on the construction of inflammatory environments and the subsequent elicitation of malignancy from an aggressive tumor type, glioblastoma (GBM). Furthermore, we show that the bioactivity of the TNIIIA2 region might become a fruitful anti-tumor target in the clinical setting.

## Biological Properties of the TNIIIA2 Region in TN-C and its Effect on Macrophages

**Figure 2:**
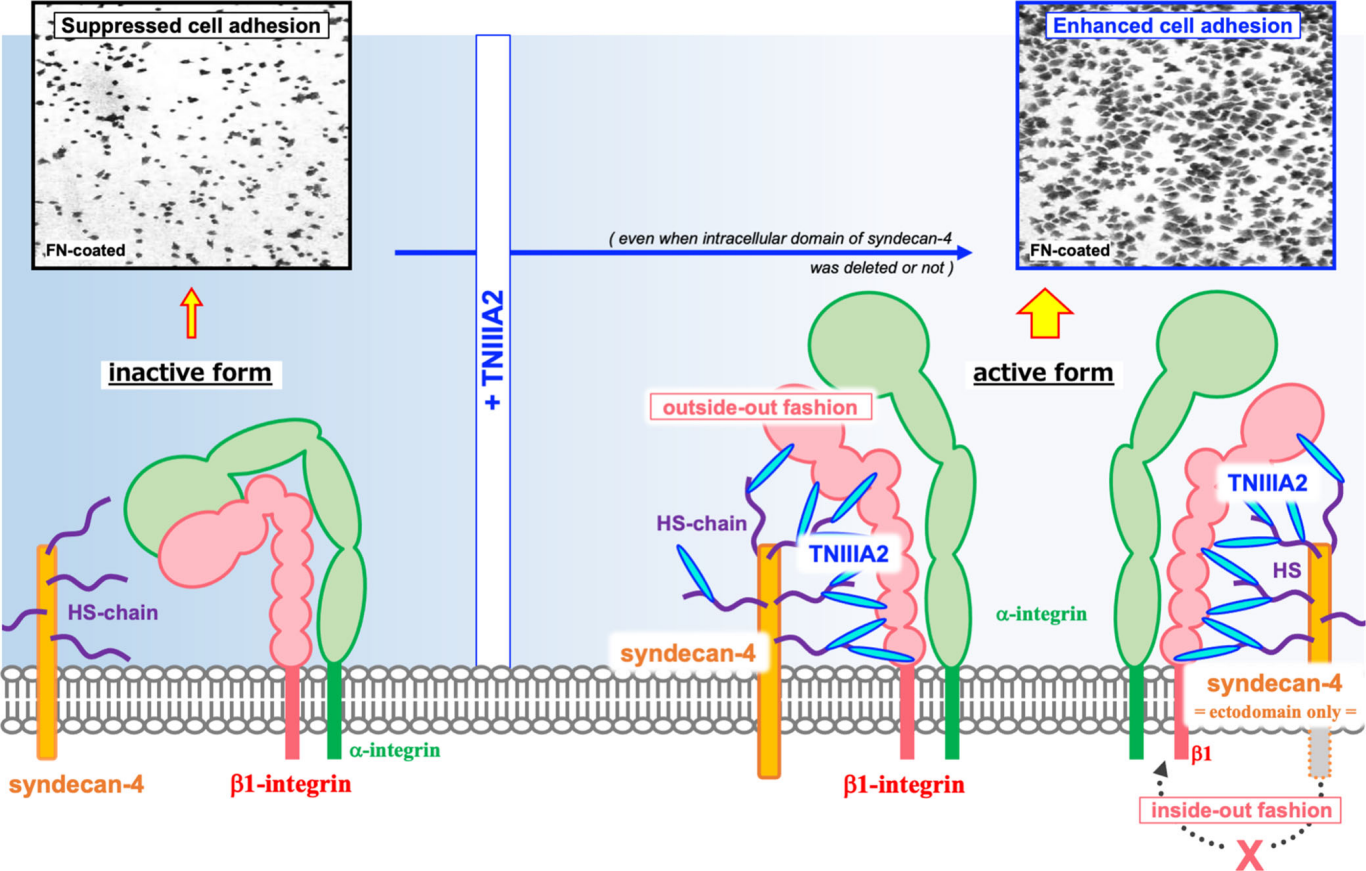
Schematic illustration of the β1 integrin activation induced by TNIIIA2. Integrin heterodimer containing β1 integrin in inactive form is shown in left side. Accompanied with the release of TNIIIA2 region from TN-C by proteolysis, TN-C fragment containing TNIIIA2 region forms complex with cell surface syndecan-4 through heparan sulfate chain. Then, conformation change of β1 integrin to active form will be occurred by lateral interaction of formed TNIIIA2/syndecan-4 complex with β1 integrin (right side). Representative image of enhanced cell adhesion induced by p-TNIIIA2 addition is shown in upper part.

The TNIIIA2 region was first identified as an accelerator of cell adhesion to FN substrate. It has been shown that WI38VA cells stimulated with a peptide containing the TNIIIA2 region (p-TNIIIA2) attached and spread rapidly to the FN substrate (13). Ability of p-TNIIIA2 to induce potentiated and sustained activation of β1 integrin has been shown (13). It has also been demonstrated that cell surface sydecan-4 is needed for the expression of pro-adhesive activity of TNIIIA2 region (13). Syndecan-4 is a member of cell surface proteoglycans that affect numerous cellular processes including growth and migration through activation of various signaling pathways. As for the relationship with β1 integrins, syndecan-4 works not only as an intracellular signal transducer but also for regulating integrin turn over expressed on cell surface and maintain focal adhesion functionality (16–21). Versatility of syndecan-4 in cell regulation depends on their ability to bind numerous molecules to their intracellular domain. Integrin turnover is also achieved by sydecan-4-induced activation of PKCα and/or ARF6 signaling pathways (16, 18). However, in our case, it was identified that the ectodomain of syndecan-4 plays an important role in β1 integrin activation in response to TNIIIA2 region because conformational change of β1 integrin was still observed even with both deletion of the cytoplasmic domains of syndecan-4 and addition of PKCα inhibitor (13). Therefore, as illustrated in **Figure 2**, it was predicted that TNIIIA2 region of TN-C binds to syndecan-4 ectodomain, nestles close to β1 integrin in inactive form, and then, forces change β1 integrin conformation to active in a lateral “outside-out” interaction (14, 15).

**Figure 3:**
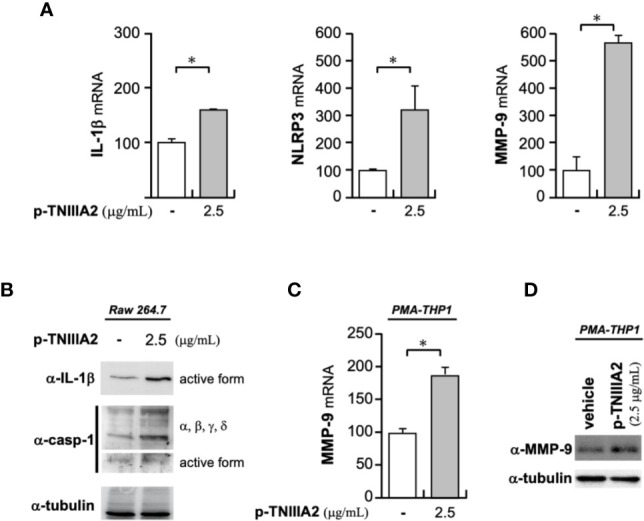
Pro-inflammatory effects of p-TNIIIA2 in macrophages. **(A)** mRNA expression in p-TNIIIA2-stimulated Raw 264.7 cells was evaluated by real-time PCR. **(B)** Western blotting analysis of IL-1β and caspase-1 in p-TNIIIA2-stimulated Raw 264.7 cells. **(C, D)** MMP-9 expression in p-TNIIIA2-stimulated THP-1 cells differentiated with PMA was evaluated by real-time PCR **(C)** and western blotting analysis **(D)**. Results are shown in the mean ± S.D. *p < 0.01 vs. p-TNIIIA2(-) control group.

Activated integrins could interact with ECM proteins, and transmit signals into cells. Intracellular signaling derived from cell adhesion receptors plays a role in regulating various cellular functions. Even in macrophages, it is generally well-known that integrin-derived signal is critical for inducing phagocytosis, cytokine release, ROS production and chemotaxis (22, 23). When we stimulated macrophage cell lines with p-TNIIIA2, upregulation of inflammatory genes including IL-1β was observed (**Figure 3A**). Western blotting analysis revealed that the upregulated IL-1β seems to be cleaved to its active form (**Figure 3B**) and is accompanied by increases in both expression and activation of caspase-1 (**Figure 3B**). Consistent with the elevation of IL-1β and caspase-1 expression in active form, NLRP3, which is a component of caspase-1-activating multiprotein complex called NLRP3-inflammasome, was also elevated in p-TNIIIA2 stimulated macrophages (**Figure 3A**). These observations suggest that the TNIIIA2 region would force macrophages to establish an inflammatory environment through elevation of IL-1β expression and following its secretion in active form. On the other hand, increased expression of TN-C, which is the parental molecule of TNIIIA2, was not detected in macrophages stimulated with p-TNIIIA2, despite the significant upregulation of MMP-9 which is needed for the release of the cryptic TNIIIA2 region from TN-C was detected (**Figure 3A**). Almost the same observation was obtained using PMA-differentiated THP-1 human macrophages (**Figures 3C, D**). These observations let us presume that macrophages have to be present in the lesion sites where parental TN-C was abundantly provided by other cells, if assuming that the macrophages stimulated by TNIIIA2 region play a role in disease progression.

## Contribution of the TNIIIA2 Region to Aberrant Cell Survival and Growth

As described above, TN-C expression in normal adult tissue is relatively low. However, transient overexpression of TN-C has been frequently observed in various types of tumor progression and the particularly high expression of TN-C in GBM, an aggressive glial tumor in adults, has been reported (24, 25). Therapeutic efficacy of anti-TN-C monoclonal antibody has been demonstrated in glioma xenograft model (26) and the positive correlation between high TN-C levels and poor prognosis in GBM patients has also been reported (5). Therefore, expression in GBM is considered a negative prognostic factor. Since, parallelism between association of proteolytic degradation of TN-C and poor prognosis in cancer including glioma (27, 28), we decided to focus on GBM next and tried to make it clear whether TNIIIA2 region, which is presumed to be released from TN-C digested by macrophage-derived MMP-9, play some roles in leading aggressive progression of tumor or not.

As described above, the function of the TNIIIA2 region is mainly based on their ability to induce conformational change of β1 integrins from inactive to active. Therefore, we first tested the effect of a β1 integrin inactivator on GBM progression *in vivo*. Fortunately, we previously identified a potent β1 integrin inactivation tool termed p-FNIII14, which is a peptide derived from 14th type III segment of FN (29). β1 integrins in active form would be forced to change their conformation to inactive form when cells were exposed to p-FNIII14 (20). We also reported that the eEF1A on cell surface act as a membrane receptor for p-FNIII14 and functional inactivation of β1 integrin was carried out by the interaction of integrins with p-FNIII14/eEF1A complex in “outside-out” fashion (30). The point is that the effect of p-FNIII14 on β1 integrin is quite the contrary to that of p-TNIIIA2 and we showed that the excessive β1 integrin activation induced by p-TNIIIA2 was able to be attenuated by addition of p-FNIII14 (31–35). When inactivation of β1 integrins was promoted in animals transplanted with GBM cells, by injection of p-FNIII14, significant suppression in the growth of GBM xenograft was observed, whereas it was continuously grown in the animals injected with biologically inactive control peptide (tumor volume on p-FNIII14 injected mice vs. control peptide injected mice = 2289.5 ± 172.5 mm^3^ vs. 5567.0 ± 779.0 mm^3^) (34). This result suggests that the malignant progression of GBM would be abrogated by β1 integrin inactivation. Because GBM cells abundantly express TN-C, MMP-mediated release of the TNIIIA2 region from TN-C appears to be a key event underlying the expression of malignancy in GBM cells, through its ability to activate β1 integrins. If so, macrophage-derived MMPs may contribute to TNIIIA2 exposure from GBM cell-derived TN-C molecules.

To investigate the TNIIIA2-mediated regulation of GBM malignancy in detail, we next evaluated the effect of p-TNIIIA2 on a GBM cell line *in vitro*. One of the characteristics of GBM cells is a high degree of proliferation and significant activation in mitogenic PDGF signaling has been reported in proneural GBM (36, 37). Given that we have previously shown that hyperstimulation of PDGF-dependent proliferation is induced when NIH3T3 cells are exposed to both p-TNIIIA2 and PDGF (14), we hypothesized that PDGF-dependent proliferation in GBM cells could also be accelerated by TNIIIA2-derived signaling. Hence, we first tested the effects of PDGF stimuli on T98G GBM cells and confirmed that T98G cell proliferation was accelerated by PDGF in a dose-dependent manner but reached a plateau at over 10 ng/ml (34). When p-TNIIIA2 was added to the culture medium of T98G cells stimulated with a submaximal dose of PDGF, the proliferation of these cells was further accelerated. The p-TNIIIA2–dependent hyperproliferation observed in PDGF-stimulated T98G cells was abrogated by the addition of function-blocking antibodies against α5 and β1 integrin, whereas anti-α4, αv, and β3 integrin antibodies showed no effect (34). We have also observed that the β1 integrin-activating antibody HUTS-4 can boost PDGF-dependent GBM cell proliferation, similar to p-TNIIIA2 (34). From these observations, PDGF-secured GBM cell proliferation is strongly stimulated further by α5β1 integrin activation, which involves binding of the TNIIIA2 region to GBM cells. TN-C, which is highly expressed in GBM lesions, would be a source of the release of TNIIIA2 region.

The effect of p-TNIIIA2 on the hyperstimulation of PDGF-dependent GBM cell growth was further proved through validation of anchorage-independent cell proliferation. As determined with a soft agar colony formation assay, the number of colonies formed was significantly elevated in T98G cells stimulated with both PDGF and p-TNIIIA2, whereas the colony number for the cells stimulated with PDGF or p-TNIIIA2 alone showed just a slight elevation (34). The β1 integrin-inactivating peptide p-FNIII14 can rescue T98G cells from an aberrant acceleration in both adhesion-dependent and -independent cell survival/growth induced by p-TNIIIA2 stimulation combined with PDGF (34). From these observations, it was suggested that the TNIIIA2 region in TN-C is responsible for inducing the hyperstimulation of GBM cell survival and proliferation, with its bioactivity inducing potent and prolonged activation of β1 integrins.

Regarding parental TN-C molecule, its ability to stimulate tumor cell growth has been reported by several groups (38–45). As a molecular mechanism for this effect of TN-C, Huang et al. demonstrated that TN-C is capable of interfering in the ligation of cell surface sydecan-4, which works as a co-receptor of integrins, to FNIII-13 domain of FN (46). They showed that the intracellular signaling pathway activated by syndecan-4 ligated to FNIII-13 domain is responsible for suppressing tumor cell proliferation (46). TN-C has ability to bind to the same FNIII-13 domain of FN. Therefore, TN-C is able to neutralize anti-proliferative signal derived from FN-ligated syndecan-4, result in vigorous cell growth accompanied by the suppression of cell adhesion in spread form (46). Consistent with this report, Orend et al. also reported that the inhibition of interaction between FNIII-13 domain of FN and sydecan-4 by TN-C leads cell cycle arrest in fibroblast (47). Moreover, inhibition of tumor cell growth by exogeneous addition of TN-C in soluble phase has been reported (48). Among these reports referred above, TN-C seems to work as an inhibitor for cell adhesion to FN. On the other hand, TNIIIA2 region acted as an inducer of cell adhesion to FN. p-TNIIIA2 could promote β1 integrin activation even when intracellular domain of its receptor syndecan-4 was deleted, and when PKCα signaling that is activated by FN-ligated sydecan-4 was inhibited (13). Since TNIIIA2 region is usually fold inside in intact TN-C molecule and exposed when TN-C was digested by inflammatory proteinase like MMPs, the mechanisms responsible for the proliferation-stimulatory effect of TNIIIA2 region seems to be different and independent from that underlying expression of TN-C’s effects at least on growth activity.

## Effect of the TNIIIA2 Region on GBM Cell Migration and Scattered Proliferation

Similar to its aggressive proliferation, GBM is also characterized by frequent dissemination throughout the brain parenchyma. Therefore, we next tested whether p-TNIIIA2 has ability to affect cell migratory and showed that the migratory activity of GBM cell lines in an *in vitro* scratch assay was significantly accelerated by p-TNIIIA2 administration (34). p-TNIIIA2-mediated elevation in cell migration activity was completely abrogated by the addition of the β1 integrin-inactivating peptide p-FNIII14 (34). Moreover, we recently reported that binding of the FN substrate to α5β1 integrin, but not α4β1 integrin, might be a key event underlying the accelerated GBM cell migration induced by p-TNIIIA2 (34). These results suggest that activation of α5β1 integrin is responsible for the augmented migratory activity observed in p-TNIIIA2–treated GBM cells.

**Figure 4:**
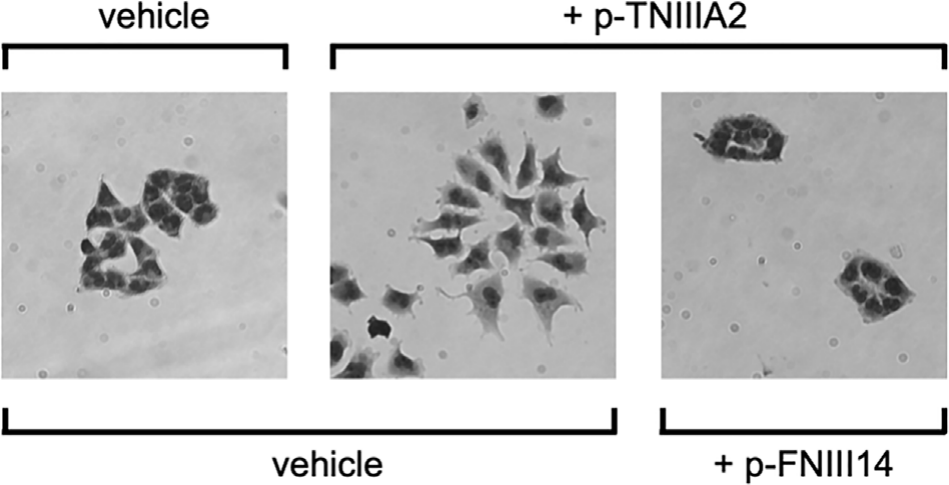
TNIIIA2-mediated acceleration of GBM cell dissemination. p-TNIIIA2-stimulated GBM cells were cultured with or without p-FNIII14. Then, the disseminative activity of these cells was evaluated by microscopic analysis.

During the experiments described above, we found that T98G cells cultured on the 2D FN substrate proliferate with a cobblestone-like cell sheet formation (**Figure 4**). Interestingly, the cell-to-cell adhesive interactions of T98G cells seemed to be arrested by the addition of TNIIIA2, resulting in a mesenchymal morphology (**Figure 4**). This “EMT-like” change induced by TNIIIA2 was completely blocked by not only a peptide that leads inactivation of β1 integrin, p-FNIII14 (**Figure 4**), but also a function-blocking antibody to β1 integrin, BV7 (34). Concomitant with the promotion of cell scattering from the cobblestone-like sheet, we also found that one of the EMT-related markers, β-catenin, was localized around the cell-to-cell contact area in GBM cells without p-TNIIIA2 stimulation, and this localization was markedly reduced and became diffuse in the cytosol when p-TNIIIA2 was added to GBM cell culture (34). These observations suggest that β1 integrin activation by the TNIIIA2 region might also be implicated in more aggressive GBM cell migration, which is one of the important characteristics of malignant disseminative behavior in GBM.

As for the parental TN-C molecule, migration-stimulatory effect has been broadly reported not only in tumor cells (4, 38, 45, 48–55) but also in non-tumor cells (56–58). Of note, Tsunoda et al. demonstrated that TN-C substrates containing FNIII-A1, -A2, -A4, -B, and -D repeat is capable of enhancing tumor cell migration whereas TN-C without alternative splicing domain showed no effect (45). They also mentioned that large variants of TN-C without FNIII-B repeat is also effective for acceleration of tumor cell migration (45). Contribution of β1-integrin in TN-C-mediated acceleration of cell migration has also been demonstrated (55, 57). Since bioactive TNIIIA2 region has been found from FNIII-A2 repeat and works as a potent inducer of β1-integrin activation, there is a possibility that the migration promotive nature of TN-C might be retained by TNIIIA2.

On the other hand, deep relationship between TN-C expression and EMT promotion has also been reported (59–65). Among them, Yoneura et al. demonstrated that the interaction of TN-C with annexin II, which is known to be able to bind to alternative splicing domain of TN-C, is capable of driving EMT and following acquisition of anoikis resistance in pancreatic cancer (61). Since TNIIIA2 region has been found from FNIII-A2 alternative splicing repeat of TN-C, and this region has ability to added anoikis resistance to GBM cell (34), the EMT-promotive effect of TN-C seems to be　expressed through exposure of TNIIIA2 region. However, Katoh et al. showed that the binding of αVβ1 and αVβ6 integrins, both of which could bind to FNIII-3 domain of TN-C, is responsible for TN-C-mediated EMT promotion in breast cancer cells (62, 63). Mamuya et al. and Tiwari et al. has also showed a contribution of FNIII-1 to 5 domains to EMT promotion in lens epithelial cells (64, 65). Thus, release of TNIIIA2 region might plays a role in EMT promotion, at least in part, depend on cellular context.

## Positive Feedback Loop Between TNIIIA2 Release and the Expression of TN-C, β1 integrin, and PDGF

**Figure 5:**
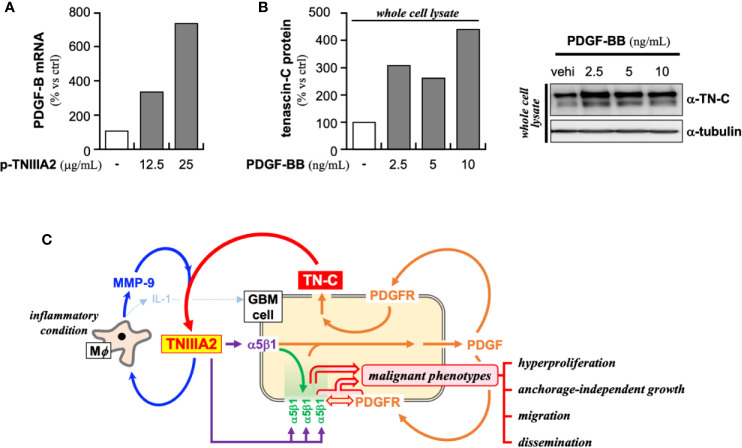
Presumed positive feedback loop in the malignant progression of GBM through TNIIIA2-derived signal transmission. **(A)** PDGF-B expression in p-TNIIIA2-stimulated GBM cells was evaluated by RT-PCR. **(B)** TN-C expression in PDGF-BB-stimulated GBM cells was evaluated by western blotting analysis. **(C)** Schematic illustration of the putative positive feedback loop underlying the acceleration of GBM aggressiveness.

In addition to the above-described experiments, we found that the PDGF-B expression in p-TNIIIA2-treated T98G cells was significantly higher than that cultured without p-TNIIIA2 (**Figure 5A**). Moreover, the expression of β1 integrin in p-TNIIIA2-treated T98G cells was also 3 to 4 times higher than untreated cells (35). Interestingly, PDGF-BB, which is upregulated in TNIIIA2-stimulated GBM cells (**Figure 5A**), can accelerate both the expression and secretion of TN-C, the TNIIIA2 parental protein (**Figure 5B**). These observations suggest that TNIIIA2 can stimulate PDGF production and that subsequent binding of PDGF to PDGF receptor on GBM cells in an autocrine/paracrine manner leads to upregulation of TN-C protein. Moreover, the TNIIIA2-derived signals would be further reinforced by TNIIIA2-mediated upregulation of β1 integrin (35), which is the molecule responsible for the bioactivity of TNIIIA2. These results suggest that there is a feedback loop between the upregulation of TN-C, TNIIIA2, β1 integrin, and PDGF (**Figure 5C**). Because MMP-9 expression in macrophages was significantly upregulated by p-TNIIIA2 at 2.5 µg/ml (**Figures 3A, C, D**), which is 10 times lower than that was needed for eliciting GBM cell malignancy, macrophages might contribute to this feedback loop and elicit the aggressive malignancy of GBM cells through promotion of the continuous release and accumulation of the TNIIIA2 region by MMP-9-mediated cleavage of TN-C, result in subsequent PDGF production from GBM cells and make this feedback loop effectively work.

## Contribution of β1 Integrin Activation to Drug Resistance

For newly diagnosed GBM patients, temozolomide (TMZ), an oral alkylating agent, is the first-line chemotherapeutic agent. However, more than half of all GBM patients show resistance to TMZ. One of the reasons for the acquisition of drug resistance in GBM is upregulation of the DNA repair protein MGMT (66). Thus, the development of new clinical strategies to overwhelm TMZ resistance is warranted. Recently, Janouskova et al. (67) showed that GBM cells with α5 integrin overexpression develop TMZ resistance and that depletion of α5 integrin sensitizes GBM cells to TMZ. α5 and β1 integrin can form a heterodimer, which would contribute to cell adhesion as a FN receptor, and we recently demonstrated that the inactivation of β1 integrins *via* p-FNIII14 administration can significantly reduce the EC_50_ of TMZ against T98G cells (34). In our experimental conditions, downregulation of MGMT appeared to be the one of underlying mechanisms for the restoration of chemosensitivity observed in FNIII14-treated GBM cells. This MGMT downregulation was not depend on the enhanced MGMT promoter methylation (34). Likewise, Wang et al. also reported a methylation independent regulation of MGMT expression (68), although the level of MGMT promotor methylation has been reported as a marker of response to TMZ in glioma (68–71). On the other hand, we have also reported previously that p-FNIII14-mediated β1 integrin inactivation can sensitize solid tumor cells to various chemotherapeutic drugs through activation of pro-apoptotic Bim (72). Taken together, although the molecular mechanisms underlying FNIII14-induced MGMT downregulation require further investigation, β1 integrin activation promoted by the TNIIIA2 region might also play a role in the acquisition of drug resistance in tumor cells.

## Discussion

In this review, we focused on GBM and proposed the existence of a positive feedback loop comprising TN-C, TNIIIA2, β1 integrin, and PDGF that can stimulate hyperproliferation, enhanced migration, and active dissemination. GBM cells can also become resistant to chemotherapeutic drugs when β1 integrin is activated. Thus, TNIIIA2-mediated potent and sustained β1 integrin activation would be a key event in eliciting the aggressive malignant phenotype of GBM. Because proliferation of GBM cells was stimulated by PDGF but reached a plateau, signals derived from PDGF binding would be insufficient to instigate the positive feedback loop mentioned above. Notably, the tumor-beneficial action of macrophages was stimulated by 2.5 µg/ml of p-TNIIIA2 whereas malignant phenotypes in GBM cell was enhanced by 25 µg/ml of p-TNIIIA2. This indicates that macrophages exposed to low levels of TNIIIA2 in the early stage of the tumor lesion establishment might contribute to the elicitation of an aggressive malignant phenotype from GBM cells *via* enhanced MMP-9 expression and subsequent digestion of TN-C and exposure of TNIIIA2 region. By the macrophage-mediated elevation in the level of the exposed TNIIIA2 region, hypothesized positive feedback loop (**Figure 5C**) can be pushed result in promotion of aggressive malignancy in GBM cells.

When focused onto the effect of parental TN-C on macrophages, accumulated evidence indicates that TN-C is capable of adding inflammatory phenotype on macrophages. Acceleration of inflammatory cytokine production promoted by FBG domain of TN-C molecule through binding to TLR4 has been shown (73, 74). Moreover, it has also been reported that the αVβ3 integrin mediated interaction of macrophages with TN-C leads production of inflammatory cytokines, such as IL-1β, IL-6, and TNF (75). However, there are no report showing MMP-2/9 upregulation in macrophages stimulated with TN-C. Moreover, both FBG and FNIII-3 domain is not defined as an alternative spliced domain. Thus, TNIIIA2-mediated polarization of macrophages toward inflammatory phenotype might be a newly acquired function of TN-C expressed through its limited digestion. In our observations, macrophages were 10 times more susceptible to p-TNIIIA2 than GBM cells. This fact let us presume that macrophages would be stimulated earlier than GBM cells during progression of tumors. If so, which cells are responsible for the “first” released of TNIIIA2 region. One of the answers to this question might be macrophages because basal expression of MMP-2 is frequently observed although it is in relatively low level. Tumor cell is also candidates because various type of tumor cells has ability to produce MMPs, especially on their way to express metastatic activity. There is a possibility that fibroblast acts as a source of MMPs, since Kanayama et al. has previously shown that MMP-9 production is upregulated when fibroblast was stimulated with FNIII-3 domain of TN-C (76). Although further examination is needed, it is supposed that the environmental MMP-2/9 level which would be drastically elevated through inflammatory activation of macrophages might be one of the key events for adding aggressive metastatic phenotypes on tumor cells.

On the other hand, as shown in **Figures 3A, B**, p-TNIIIA2 is able to stimulate IL-1β production in macrophages. IL-1β has been reported to be the factor that makes the tissue environment favorable for solid tumor progression through the acceleration of tumor cell growth, invasion, and neovascularization (77–79). In addition, regarding GBM progression, Paugh et al. reported that IL-1 can boost GBM cell survival and invasion through sphingosine kinase 1 upregulation (80). Moreover, we recently reported that the acceleration of survival and proliferation in preneoplastic cells was induced by soluble paracrine factors secreted from TNIIIA2-stimulated fibroblasts (33). Furthermore, we have also reported that TNIIIA2 is able to increase the invasive and metastatic activity of colon cancer cell lines (81). The contribution of β1 integrin activation to the acquisition of drug resistance is not limited to GBM cells but broadly observed in various solid tumor cell lines, although the suggested underlying mechanisms differ between GBM and other cell types (34, 72). These observations led us to presume that the released TNIIIA2 region might be able to elicit malignant phenotypes from tumor cells universally. The macrophage-mediated splicing of TN-C seems to be a key event for eliciting aggressive malignant phenotypes from tumor cells.

In any case, the accumulated evidence suggests that the TNIIIA2 region in TN-C might be a powerful initiator for the elicitation of aggressive properties from tumors through potentiated and sustained activation of β1 integrins. Macrophages, which are present in or infiltrate into the tumor stroma and can react to TNIIIA2 region at low levels, play a key role in ensuring that the level of released TNIIIA2 region is sufficient to promote the aggressive properties of tumors. Therefore, inactivation of β1 integrin would provide a useful strategy to abrogate the acquisition of aggressive behaviors by tumors, including GBM. The combined administration of the peptide FNIII14 and chemotherapeutic drugs would be a promising strategy for clinical cancer treatment. Actually, Matsunaga et al. previously reported that β1 integrin-mediated adhesion of AML cells is crucial for the formation or minimal residual disease at bone marrow which is the major cause of relapse after chemotherapy in AML patient (82) and showed the therapeutic efficacy of combined administration of β1 integrin inactivator p-FNIII14 with chemotherapeutic drug to eradicate MRD and suppress AML relapse in mice (83). Although further investigation and more detailed information is needed, TNIIIA2 region itself of releasing mechanism of this region might become a fruitful target for establishing new therapeutic strategies targeting TN-C.

In addition, as described in this review, since TNIIIA2 region was found as a potent and sustained activator of β1 integrin, and since β1 integrin-mediated cell adhesion is broadly observed in various types of cells, it is likely that the pathological and physiological properties of TNIIIA2 region would not be restricted to tumors. Actually, investigations examining the role of TNIIIA2 region in other pathological conditions are being performed by several groups lately (84–87). Further advances in the research focused on TNIIIA2 region are expected and would be of interest.

## Author Contributions

TI and MF wrote, reviewed, and proofread the manuscript. FF reviewed and proofread the manuscript. All authors contributed to the article and approved the submitted version.

## Conflict of Interest

The authors declare that the research was conducted in the absence of any commercial or financial relationships that could be constructed as a potential conflict of interest.
